# Sp1 Expression Is Disrupted in Schizophrenia; A Possible Mechanism for the Abnormal Expression of Mitochondrial Complex I Genes, *NDUFV1* and *NDUFV2*

**DOI:** 10.1371/journal.pone.0000817

**Published:** 2007-09-05

**Authors:** Dorit Ben-Shachar, Rachel Karry

**Affiliations:** 1 Laboratory of Psychobiology, Department of Psychiatry, Rambam Medical Center, Ruth and Bruce Rappaport Faculty of Medicine, Technion Israel Institute of Technology, Haifa, Israel; 2 Laboratory of Molecular Medicine, Rambam Medical Center, Ruth and Bruce Rappaport Faculty of Medicine, Technion Israel Institute of Technology, Haifa, Israel; Chiba University, Japan

## Abstract

**Background:**

The prevailing hypothesis regards schizophrenia as a polygenic disease, in which multiple genes combine with each other and with environmental stimuli to produce the variance of its clinical symptoms. We investigated whether the ubiquitous transcription factor Sp1 is abnormally expressed in schizophrenia, and consequently can affect the expression of genes implicated in this disorder.

**Methodology/Principal Findings:**

mRNA of Sp1 and of mitochondrial complex I subunits (*NDUFV1*, *NDUFV2*) was analyzed in three postmortem brain regions obtained from the Stanley Foundation Brain Collection, and in lymphocytes of schizophrenic patients and controls. Sp1 role in the transcription of these genes was studied as well. Sp1 was abnormally expressed in schizophrenia in both brain and periphery. Its mRNA alteration pattern paralleled that of *NDUFV1* and *NDUFV*2, decreasing in the prefrontal cortex and the striatum, while increasing in the parieto-occipital cortex and in lymphocytes of schizophrenic patients as compared with controls. Moreover, a high and significant correlation between these genes existed in normal subjects, but was distorted in patients. Sp1 role in the regulation of complex I subunits, was demonstrated by the ability of the Sp1/DNA binding inhibitor, mithramycin, to inhibit the transcription of *NDUFV*1 and *NDUFV*2, in neuroblastoma cells. In addition, Sp1 activated *NDUFV2* promoter by binding to its three GC-boxes. Both activation and binding were inhibited by mithramycin.

**Conclusions/Significance:**

These findings suggest that abnormality in Sp1, which can be the main activator/repressor or act in combination with additional transcription factors and is subjected to environmental stimuli, can contribute to the polygenic and clinically heterogeneous nature of schizophrenia.

## Introduction

Schizophrenia is known for its great heterogeneity across individuals and variation in symptoms within individuals over time [1], [2]. Based on family, twin, and adoption studies, it is accepted that genes play a major role in the susceptibility for schizophrenia. Like most other complex disorders, schizophrenia is currently believed to be a polygenic disease. Molecular studies in schizophrenia, mostly in post mortem brains, have unraveled abnormalities in a variety of functional groups of genes. For example, reduced expression was observed in genes associated with synaptic architecture and transmission including the presynaptic vesicle proteins such as the synapsins [3], the SNARE vesicle-docking complex and synaptosomal-associated protein-25 (SNAP-25) [4], [5]. In addition, reduced expression of synaptic proteins such as neuregulin 1 [6], dysbindin, regulator of G-protein signaling 4 (RGS4) [6], [7], and of reelin, which is secreted by GABAergic neurons in association with dendritic specializations [8], were reported. Abnormalities were also observed in genes associated with metabolism and/or receptors of schizophrenia-relevant neurotransmitters including dopamine (DA) (DA D3/D4 receptors, COMT) GABA (GAD67, GABA(A) receptor) and glutamate (GLUR1/2, NMDA NR1/2, PSD proteins) [9], [10]. Recently, a robust change was demonstrated in genes and proteins associated with mitochondria in schizophrenia, including those encoding the oxidative phosphorylation systems (OXPHOS) [11]–[13]. It is therefore currently hypothesized that in complex disorders such as schizophrenia, susceptible genes can combine with each other and with environmental modifiers as well as with other modulating unaffected genes to produce the variance in the syndromal picture of the disorder.

We further hypothesize that an abnormality in a ubiquitous transcription factor that participates in the regulation of numerous genes, either as the main activator/repressor or in combination with additional transcription factors, all differentially subjected to environmental stimuli, can lead to the heterogeneity observed in schizophrenia. The transcription factor Specificity protein 1 (Sp1), is such a candidate. Sp1 is the prototype of a family of zinc finger (Cys_2_/Hys_2_) DNA binding transcription factors that binds to and acts through G-rich elements such as GC-box. It is generally accepted that this extremely versatile protein is involved in the expression of many different genes and can be regulated at the level of transcription and post-translational modifications such as phosphorylation and/or glycosylation [14]. Among the various factors that can modulate Sp1 activity are glucose, insulin and several kinases including CDK2 and ERK1/2 [14]–[17]. Sp1 can form homotypic interactions as well as heterotypic interactions with different classes of transcription factors including the TATA-box binding protein (TBP), TPB-associated factors (TAFs) and Sp3 [14], [18]. Interestingly, Sp1 is involved in the regulation of several genes which have been implicated in schizophrenia such as reelin, GAD67, MAOA/B, NMDA receptor subunits NR1 and NR2A/B, GABA A and DA receptors D1A and D2/3 [19]–[24]. In addition, Sp1 is involved in the regulation and coordination of nuclear encoded mitochondrial genes including some which encode for OXPHOS proteins [25], [26]. In line with the latter, it is noteworthy that GC-box binding sites for Sp1 are found in most, if not all, OXPHOS promoters characterized to date, and in several of them are the only putative binding sites [25].

Accumulating data points to abnormalities in the OXPHOS enzyme activity as well as mRNA and protein expression in schizophrenia both in periphery and in brain [11], [12], [27]–[32]. We have previously reported disease specific and state dependent alterations in complex I activity, which was shown to play a major role in controlling oxidative phosphorylation in synaptic mitochondria [33], in lymphocytes, and platelets of schizophrenic patients. These changes were associated with region specific alterations in postmortem brain and in periphery, of mRNA and protein expression of three complex I subunits, the 24kDa *(NDUFV2*), the 51kDa *(NDUFV1)* and the 75kDa *(NDUFS1)* subunit, all forming one functional subunit [34].

Herein we show for the first time that in schizophrenia Sp1 mRNA levels are altered in three different brain areas and in the periphery in a region specific manner. Moreover, Sp1 changes parallel the tissue specific pattern of change in the expression of *NDUFV1* and *NDUFV2* in all four tissues. In addition, a high and significant correlation between these genes is observed in normal subjects but is distorted in patients. We further show that the inhibition of Sp1 binding to DNA, by mithramycin, modulates the expression of *NDUFV2, NDUFV1*, *NDUFS1* and *RELN* (reelin), all reported to be altered in brains of schizophrenic patient. Moreover, the TATA-less 5′-flanking sequence of the human *NDUFV2*, which was the most affected subunit of complex I in schizophrenia, demonstrated a promoter activity. This promoter sequence contains three regulatory elements that were recognized by Sp1. These results suggest Sp1 as a pathological factor in schizophrenia, which may explain the multi-gene manifestation of this disorder.

## Materials and Methods

### Subjects

Ten inpatients who met DSM-IV criteria for schizophrenia and had pronounced positive symptoms (PANSS scores ≥ 20) participated in the study. Consensus diagnosis by two senior psychiatrists was based on extended clinical interview and patients' chart review. Severity of symptoms was evaluated using the Positive and Negative Symptom Scale (PANSS) for schizophrenia and the Clinical Global Impression (CGI) scale. All patients were treated with antipsychotic medications. Patients with schizoaffective illness were excluded. Ten age and sex matched subjects without prior psychiatric history served as controls. Excluded from the study were subjects suffering from a severe current medical condition, present or past neurological disorder, history of head trauma with loss of consciousness greater than 10 minutes or any medical condition requiring somatic medication. All subjects were given an explanation on the purpose of the study and provided a written informed consent. The study was approved by The Rambam Medical Center Institutional Review Board.

### Isolation of platelets and lymphocytes

Blood (20 ml) was collected from the cubital vein without tourniquet between 8.00 and 10.00AM. Platelets were isolated from platelet-rich plasma (1–2 hr after blood sampling) by centrifugation at 1000×g for 30 min. Lymphocytes were separated on Ficoll-Plaque gradients by centrifugation at 400×g for 30 min. Platelets or lymphocytes were immediately further processed and total RNA was isolated

### Post-mortem tissues

Frozen samples from the prefrontal cortex, including the middle frontal gyrus of the frontal lobe (BA46/9), from the ventral parieto-occipital cortex (BA19) and from the striatum including a part of the nucleus accumbance, were provided by the Stanley Foundation Neuropathology Consortium (Bethesda, MD). Samples were obtained from individuals diagnosed with schizophrenia (DSM-IV criteria, n = 15) and from normal controls (n = 15). The groups were matched by age, sex, race, postmortem interval (PMI), pH, side of brain, and mRNA quality. Demographic data are presented in Table 1. A description of the Stanley Brain Collection and more detailed demographics and samples' quality were previously reported [35]. All samples were analyzed in parallel, blind to patients' diagnosis. Protection of human rights and detailed information regarding the selection of specimen can be found in [35]. In short, for all specimens a pathologist contacted the family of the deceased to make a preliminary diagnosis and requested permission for donation of the brain and for the release of the deceased's medical records. No name or other identifying information was given for the deceased.

**Table 1:** Demographic data for post mortem brains.

Variable	Control (n = 15)	Schizophrenia (n = 15)
Age (years, means±SD)	48.1±10.7	45.57±12.95
Gender (male, female)	9M, 6F	8M, 6F
Postmortem interval (h, means±SD)	23.7±9.94	33.79±15.16
Cause of death		
*Cardiac*	13	6
*Accident*	2	2
*Suicide*		4
*Other*		3
Age of onset (years, means±SD)	N/A	23.93±7.72
pH (means±SD)	6.3±0.2	6.2±0.25
Brain hemisphere used (right∶left)	8∶7	9∶5
Lifetime antipsychotic dose^a^ (mg, means±SD)	0	52428±64401
*Typical*		5
*Atypical*		3
*Both*		3
*None*		3
History of psychosis	0	14
Current alcohol/drug abuse or dependence	0	3
Past alcohol/drug abuse or dependence	2	3

a Lifetime antipsychotic dose in fluphenazine milligram equivalents. N/A–not applicable

### Cell culture

SH-SY5Y or SK-N-SH human neuroblastoma cells were grown at 37°C, with 5% CO2 in Dulbecco's modified Eagle's Medium (DMEM) containing 4.5 gm/l D-Glucose and supplemented with 2 mM L-glutamine, 100 U/ml streptomycin/penicillin and 10% FCS. To inhibit Sp1-DNA binding, cells were grown in the presence of 100–400 nM mithramycin in serum free medium for 12–48 hrs. Cell viability was determined by Trypan Blue. Mithramycin at concentration<200 nM for up to 36 hrs had no effect on cell viability. The following experiments were performed in the presence of 150 nM mithramycin for 24 hrs.

### RNA extraction

RNA extraction from blood cells or tissue was performed using RNA STAT-60 kit (TEL-TEST, INC, Frienwood, TX, USA). For further purification of RNA, the phenol∶chloroform∶isoamyl alcohol (25∶24∶1) step was performed twice. All RNA preparations were dissolved in RNase free water and were then treated by DNase as previously described [29]. The final RNA preparation was dissolved in RNase free water and stored in −80°C until use. RNA integrity, depicted in the form of three bands corresponding to 28S, 18S and 5S RNA, was assessed by electrophoresis in a 1% agarose/formaldehyde gel stained with ethidium bromide. The amount of RNA extracted was determined spectophotometricaly at 260 nm.

### RT-PCR analysis

The expression of the different genes was studied by using RT-PCR[29]. 5 µg of total RNA were reverse transcribed using Moloney Murine Leukemia Virus (M-MLV) reverse transcriptase (Promega, Madison, WI, USA). Amplification of RT-cDNA was initially performed on a control specimen at different concentrations to define a linear range for all genes. The number of cycles, cDNA amount and primers concentration was established according to a stringent calibration process determining the log-linear phase of amplification for each gene. After establishing the optimal reaction conditions, PCR amplification was performed at least twice per individual. PCR products were stained with ethidium bromide and analyzed by electrophoresis on 2% agarose gel. Results were analyzed by densitometer. Sequences of PCR primers for *NDUFV2, NDUFV1 NDUFS1*, *RELN* , Sp1, 18S-RNA and β-actin were designed according to sequence obtained from Gene-Bank (Table 2). β-actin and 18S-RNA were used for assessment of RNA quality and yield. The β-actin was used for normalizing variations in RNA aliquots taken for RT reactions. A single batch of RNA isolated from human platelets, on which PCR was performed at three different concentrations, was assayed in parallel with each set of samples and served as a positive control and to ensure linearity. All subject samples were further normalized to this positive control to enable comparison between samples.

**Table 2:** Primer sequences and PCR conditions.

mRNA		primer sequence	Denaturing temperature and time °C (s)	Annealing temperature and time °C (s)	Elongation temperature and time °C (s)	Number of cycles	Product size (bp)
*NDUFV2*	S	5′-GGAGGAGCTTTATTTGTGCAC-3′	94 (60)	55 (60)	72 (60)	35	640
	NS	5′-CCTGCTTGTACACCAAATCC-3′					
*NDFUV1*	S	5′-TACATCCGAGGGGAATTCTACA-3′	94 (60)	60 (60)	72 (60)	35	426
	NS	5′-GTTCTTTCAAGGGCACAGACAT-3′					
*NDUFS1*	S	5′-TACTCGCTGCATCAGGTTTG-3′	94 (60)	58 (60)	72 (60)	35	299
	NS	5′-CATGCATACGTGGCAAAATC-3′					
Sp1	S	5′-GGAGAGCAAAACCAGCAGAC-3′	95 (60)	60 (30)	72 (90)	35	335
	NS	5′-CAATGGGTGTGAGAGTGGTG-3′					
Reelin	S	5′-ATGTGGTAAAGGCGTTCCTG-3′	95 (60)	58 (30)	72 (90)	35	382
	NS	5′-GGCCTTTTCAATGAAGACCA-3′					
β-actin	S	5′-TGAAGTGTGACGTGGACATCCG-3′	94 (60)	60 (60)	72 (60)	25	447
	NS	5′-GCTGTCACCTTCACCGTTCCAG-3′					
18S-RNA	S	5′-AGGAATTGACGGAAGGGCAC-3′	94 (60)	60 (60)	72 (60)	25	324
	NS	5′-GTGCAGCCC CGGACATCTAAG-3′					

All templates were initially denatured for 5 min at 94°C, and after completing all cycles, were extended a final extension of 10 min at 72°C.

### Promoter luciferase constructs

PCR was performed to generate the construct of the *NDUFV2* predicted promoter (a 461 bp genomic fragment of the 5′-flanking sequence upstream to its published translation initiation ATG), using a set of specific primers for the human sequence, 5′-GCACACTGGATAGCAGCCCTCTG-3′ and 5′-GGCGGGCCACACTGTTCACCTTC-3′. The PCR blunted-end product was subcloned into pSTBlue1 acceptor vector, using the Acceptor Vector kit (Novagen, EMD Biosciences, US), digested with kPN1 and SacI and subcloned into kPN1/SacI sites of the reporter vector pGL3 basic. Each of the constructs was analyzed by both PCR and sequencing following digestion with the relevant restriction enzymes.

### Transient transfection and luciferase assay

Transient transfections of SH-SY5Y or SK-N-SH cells with 0.5–2 µg plasmid DNA were carried out using FuGENE 6 (1∶6 w∶v) (Roche Diagnostic Hoffmann-La Roche Ltd, Basel, Switzerland). Luciferase assay was performed 12, 24 and 36 hrs after transfection with optimal results at 24 hr after transfection, using Luciferase assay kit (Promega Co. US). Luciferase activity was measured by microplate luminometer (Anthos Lucy 1, Antos Labtec Instruments, Salsburg, Austria). Cells transfected with the insert or empty pGL3 basic served as a control. Transfected cells were also incubated in the presence of 150 nM mithramycin, for 12, 24, 36 hrs, and subjected to the luciferase assay. To control for mithramycin non-specific activity, cells were transfected with pTAL-Luc and pAP1-Luc, which contains TATA-like promoter and four tandem copies of the AP1, respectively.

### Nuclear extract preparation and electrophoretic mobility shift assay (EMSA)

Nuclear extracts were prepared from SH-SY5Y and SK-N-SH cells as previously described [36]. For EMSA, three different double stranded oligonucleotides, each containing a Sp1 consensus binding site, were synthesized from the predicted promoter area of the *NDUFV2*, and radiolabeled with [γ^32^P]-ATP, using polynucleotide kinase. The forward oligonucleotides for *NDUFV2* were 5′-GGACTGGTCCCCGCCCCTCCCCCGGGAAG-3′, 5′-GGGAAGTCTCCCGCCCACAGGGCCCCAGC-3′ and 5′-CCGTCAGCCCCCGCCCCTCGGCGAA GG-3′. For EMSA, 3 µg nuclear extract proteins were incubated with 0.5 ng of the synthesized or 0.1 ng of the commercial (Promega Co. Madison WI, US) [γ^32^P]-ATP end-labeled double strand oligonucleotide, containing the consensus sequence for Sp1, for 20 min at room temperature in binding buffer (10 mM Tris-HCl pH-8, 150 mM KCl, 0.5 mM EDTA, 0.1% Triton-X 100, 12.5% glycerol (v/v), 0.2 mM DTT) in the presence of 1 µg poly-dIdC. In competition experiments, a 100 fold molar excess of unlabeled double stranded oligonucleotides or mithramycin 150 nM were added for 10 min at room temperature before the addition of the ^32^P-labeled oligonucleotides. For supershift analysis, 1 µg of Sp1specific antibody (sc-59 X, Santa Cruz Biotechnology, Santa Cruz CA) was preincubated with nuclear extract proteins for 1 hr at 4°C before the addition of the DNA probes. DNA-protein complexes were separated by electrophoresis in 5% polyacrylamide gel. Gels were dried and visualized by autoradiography.

### Statistical analysis

Results were analyzed for normal distribution using Kolmogorov-Smirnov test. Normally distributed data were analyzed by one-way ANOVA followed by post-hoc Dunnett test with comparison to the control group. Data from postmortem brains that did not distributed normally were analyzed by non-parametric Wilcoxon Mann Whitney test. Differences in means between groups were considered significant if p<0.05. For postmortem brain results, age, gender, laterality, PMI, brain pH, duration of disease and medication were added as covariates and the persistence of a significant difference in main effect between diagnostic groups was assessed by ANCOVA. Pearson's correlation test was used for the correlations between the expressions of all three genes, which show normal distribution. SPSS version 14.0 software was used for statistical analyses.

## Results

### Sp1 expression is altered in schizophrenia and parallels the region specific changes in complex I subunits, *NDUFV1* and *NDUFV2*

**Figure 1:**
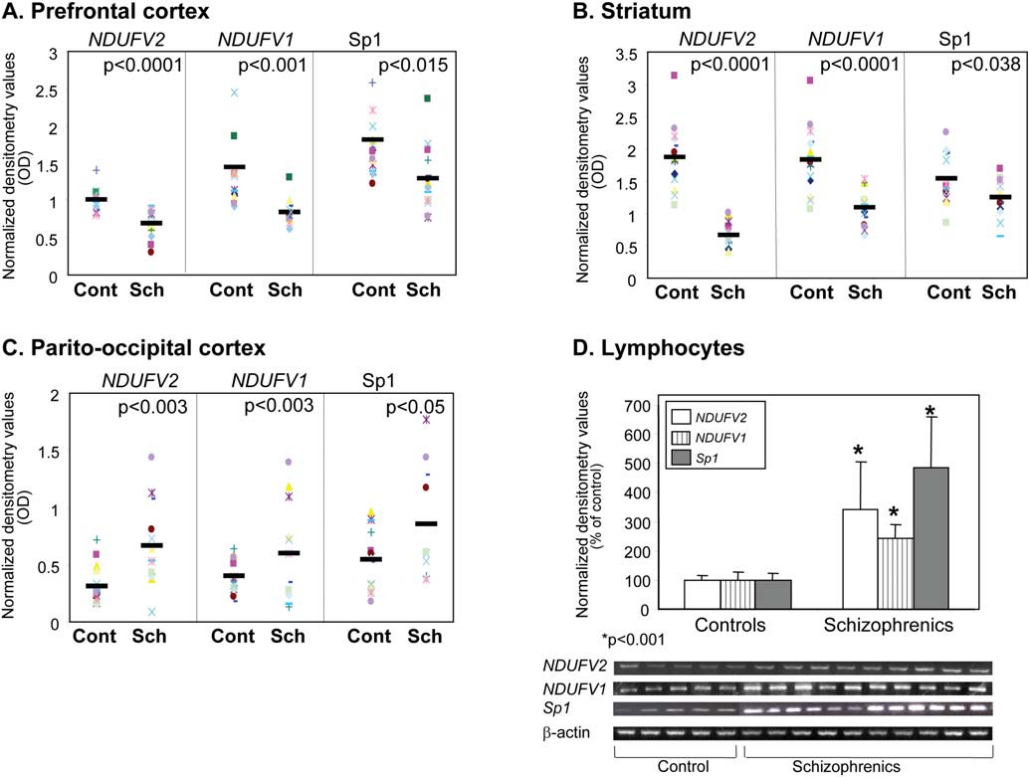
mRNA levels of *NDUFV, NDUFV2* subunits of complex I and of Sp1 in post mortem brain specimens and lymphocytes of schizophrenic patients and healthy controls. RNA was extracted from postmortem brain specimens of schizophrenic patients (SCH n = 15) and healthy subjects (Cont n = 15), and from lymphocytes of 10 inpatients and 10 healthy subjects. A. Prefrontal cortex (BA 46/9), B. Striatum (including nucleus accumbens), C. Ventral parieto-occipital cortex (BA19) and D. Densitometry values and a representative gel of mRNA levels in Lymphocytes.

Previously, we have shown that in schizophrenia the mRNA and protein expression of the *NDUFV1* and *NDUFV2* were increased in platelets and lymphocytes [29] as well as in the ventral parieto-occipital cortex, while reduced in the prefrontal cortex [13] as compared with healthy subjects. In this study, we repeated these findings using additional samples from the prefrontal and parieto-occipital cortices, and expanded them by including specimens of the striatum and lymphocytes. A significant reduction was observed in mRNA levels of *NDUFV1* and *NDUFV2* in the prefrontal cortex (67% p<0.001 and 48% p<0.0001 of control, respectively) and the striatum (60%p<0.0001, 37% p<0.0001 of control, respectively) (Fig 1A,B). In contrast, mRNA levels of *NDUFV1* and *NDUFV2* were significantly increased in the ventral parieto-occipital cortex (165%, p<0.03 and 208%, p<0.003 of control, respectively) and in lymphocytes (242% and 342%, p<0.0001 of control, respectively) (Fig 1C,D). Sp1 mRNA expression was also altered in brain and in lymphocytes in schizophrenia (Fig 1). Thus, Sp1 mRNA levels were significantly decreased in the prefrontal cortex (66%, p<0.015) and in the striatum (80%, p<0.038), while increased in the ventral parieto-occipital cortex (158%, p<0.05 of control) and in lymphocytes (480%, p<0.0001), showing region dependent changes parallel to that of complex I subunits.

In order to further study the relationship between the two subunits of complex I and Sp1, Pearson's correlation was performed in all three brain regions the prefrontal cortex, the parieto-ocipital and the striatum as well as in lymphocytes of normal subjects and schizophrenic patients (Table 3). In normal subjects, Sp1 showed a significant high correlation with both subunits of complex I in all three brain regions and in lymphocytes, except for the *NADUFV1* in the parieto-ocipital. However, in patients, there was no significant correlation between Sp1 and both genes in brain and lymphocytes, except for *NDUFV2* in the parieto-ocipital, and *NDUFV1* in the prefrontal cortex. Pooling all three-brain regions together demonstrated a highly significant correlation between Sp1 and both genes in normal subjects, while in schizophrenic patients there was no correlation with *NADUFV2*, and Sp1 correlation with *NADUFV1* was significantly reduced. Interestingly, the significant high correlation that was observed between *NDUFV1* and *NDUFV2* in normal subjects in all tissues examined, disappeared in schizophrenic patients.

**Table 3:** Pearson's correlation between *NDUFV1, NDUFV2* and *Sp1* mRNA expression in brain and lymphocytes of schizophrenic and normal subjects.

Group	Tissue		*NDUFV2*		*Sp1*	
			Pearson Correlation	Significance (p)	Pearson Correlation	Significance (p)
**Normal**	**Fcx, P-O, Str**	*NDUFV1*	0.833	0.000	0.746	0.000
		*NDUFV2*			0.559	0.000
**Schizophrenia**	**Fcx, P-O, Str**	*NDUFV1*	0.224	0.158	0.473	0.002
		*NDUFV2*			0.245	0.122
**Normal**	**Fcx**	*NDUFV1*	0.794	0.000	0.642	0.01
		*NDUFV2*			0.460	0.05
**Schizophrenia**	**Fcx**	*NDUFV1*	0.269	0.353	0.614	0.019
		*NDUFV2*			0.141	0.631
**Normal**	**P-O**	*NDUFV1*	0.760	0.002	0.347	0.269
		*NDUFV2*			0.561	0.04
**Schizophrenia**	**P-O**	*NDUFV1*	0.549	0.1	0.431	0.214
		*NDUFV2*			0.614	0.034
**Normal**	**Str**	*NDUFV1*	0.987	0.000	0.636	0.019
		*NDUFV2*			0.644	0.018
**Schizophrenia**	**Str**	*NDUFV1*	0.126	0.668	0.412	0.162
		*NDUFV2*			0.299	0.452
**Normal**	**Lymphocytes**	*NDUFV1*	0.973	0.000	0.970	0.000
		*NDUFV2*			0.944	0.001
**Schizophrenia**	**Lymphocytes**	*NDUFV1*	0.340	0.356	0.163	0.562
		*NDUFV2*			0.480	0.070

Pearson's correlation was performed across three brain regions the prefrontal cortex (Fcx) the parieto-occipital cortex (P-O), the striatum (Str) and Lymphocytes.

To control for potential confounds, age, gender, PMI, brain pH, side of brain, duration of disease and psychotropic medication (lifetime antipsychotic dose expressed in fluphenazine milligram equivalents) were added as covariates, and assessed by ANCOVA for all three genes in all brain regions. Disease related significant differences, and adjusted fold changes in mRNA after ANCOVA analysis for all three genes in schizophrenia and controls are presented in Table 4. None of the covariates could account for the significant differences between groups. Following ANCOVA analysis, the difference between groups remained significant for all genes in all brain regions, except for Sp1in the striatum. In this brain region none of the covariates had a significant effect on group differences, except for PMI (p = 0.012) which did not show a significant correlation with Sp1 expression (r = 0.257; p = 0.17).

**Table 4:** The influence of covariates on disease related significant differences in mRNA of *NDUFV1, NDUFV2* and *Sp1*in the prefrontal and the parieto-occipital cortices and the striatum.

		Adjusted fold changes in mRNA Schizophrenia/Normal	Significant group difference (p)
**Prefrontal Cortex**	*NDUFV1*	0.61	0.000
	*NDUFV2*	0.67	0.029
	*Sp1*	0.7	0.049
**Striatum**			
	*NDUFV1*	0.37	0.000
	*NDUFV2*	0.43	0.000
	*Sp1*	0.89	NS
**Parieto-occipital cortex**			
	*NDUFV1*	3.08	0.002
	*NDUFV2*	1.72	0.050
	*Sp1*	1.97	0.005

Data of ANCOVA analysis. Age, gender, PMI, brain pH, side of brain, duration of disease and psychotropic medication were included as covariates

### Mithramycin modulates mRNA expression of *NDUFV2, NDUFV1 NDUFS1* and *RELN* in SH-SY5Y cells

**Figure 2:**
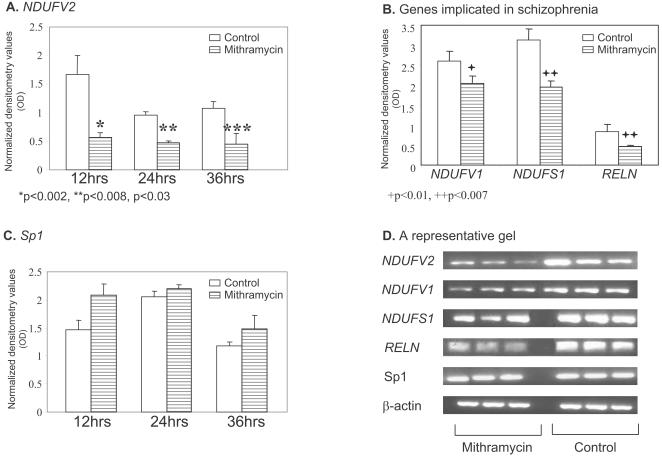
Inhibition of the transcription of *NDUFV1, NDUFV2* and *NDUFS1* subunits of complex I and of Sp1 and *RELN* by mithramycin in human neuroblastoma SH-SY5Y cells. Cell were treated for 12, 24 and 36 hrs with 150 nM mithramycin and mRNA levels of all 5 genes were analyzed by RT-PCR. A. *NDUFV2*. B. *NDUFV1* and *NDUFS1* and *RELN* after 24 hrs treatment with mithramycin. C. Sp1 and D. A representative gel, demonstrating mithramycin inhibition of the expression of the three subunits of complex I and of *RELN* with no change in Sp1 and β-actin. Results are means±SD of 3–4 experiments.

In order to assess whether Sp1 is involved in the regulation of the expression of complex I subunits, we treated SH-SY5Y cells with mithramycin (150 nM). Mithramycin inhibited mRNA expression of three subunits of mitochondrial complex I, *NDUFV2, NDUFV1* and NDUFS1 as well as that of *RELN* in a time dependent manner (Fig 2). Already after 12 hrs mithramycin administration induced a marked reduction (73%, p<0.002) in mRNA levels of *NDUFV2*. Following 24 hours of treatment a smaller but significant reduction in the expression of *NDUFS1* and *RELN* (30%, p<0.007 and 44%, p<0.007, respectively), and a mild decrease in mRNA levels of *NDUFV1* (25% p<0.01) was also observed (Fig 2B). No significant time dependent changes were observed in Sp1 mRNA levels as well as in β-actin levels (Fig 2 C,D).

**Figure 3:**
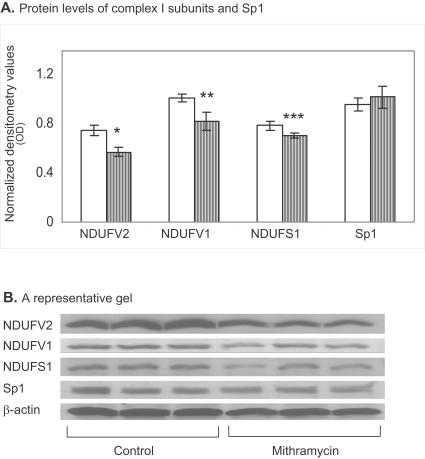
Protein levels of *NDUFV1, NDUFV2* and *NDUFS1* subunits of complex I, but not that of Sp1 are reduced following treatment of SH-SY5Y cells with mithramycin. Densitometry value of protein levels (A) and a representative gel (B) of all 4 genes in cells treated for 24 hrs with 150 nM mithramycin. Results are means±SD of 3–4 experiments. *p<0.005 **p<0.014 ***p<0.024 vs. untreated control cells.

The inhibitory effect of mithramycin on protein levels of the three subunits of complex I, the 24- 51- and 75-kDa encoded by *NDUFV2, NDUFV1 NDUFS1*, respectively, was delayed and less prominent compared to its effect on the transcription of these genes. A mild, but significant, reduction of 23% p<0.005, 20% p<0.01 and 10% p<0.02 in the 24- 51- and 75-kDa subunits, respectively, was observed 24 hrs after administration of mithramycin with no change in Sp1 protein levels (Fig 3). Similar results were observed using the SK-N-SH human neuroblastoma cell line (data not shown).

### Transcriptional activity of *NDUFV2* 5′-flanking region and its modulation by Sp1

**Figure 4:**
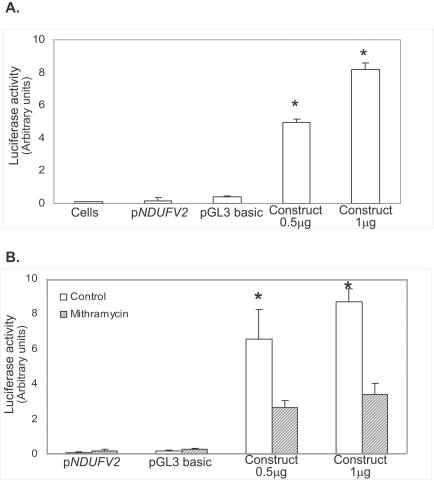
The effect of mithramycin on the transcriptional activity of the *NDUFV2* predicted promoter in SH-SY5Y cells. A. Cells were transiently transfected with two concentrations of the p*NDUFV2*-Luc reporter construct (construct) for 24 hrs and analyzed for luciferase activity. B. One hour before the transfection cells were pre-incubated with or without mithramycin (150 nM). The results are means±SD of three experiments normalized for10^6^ cells. *p<0.0001.

In our previous studies, out of the three complex I subunits, *NDUFV2* showed the greatest abnormality in schizophrenic patients [13], [29]. In addition, the present study demonstrate that *NDUFV2* transcription was strongly affected by mithramycin in SH-SY5Y and SK-N-SH cells, suggesting an important regulatory role for Sp1 transcription factors in its expression. Therefore, we used this gene to further study the role of Sp1 in its transcription. First, we tested whether the 461 bp genomic fragment of the 5′-flanking sequence upstream to *NDUFV2* published translation initiation ATG has promoter activity by subcloning into a luciferase reporter plasmid and transfecting SH-SY5Y or SK-N-SH cells. As predicted, cells transfected with the construct containing the 461 bp genomic fragment of *NDUFV2* demonstrated a dose dependent increase in luciferase activity as compared to control cells transfected with pGL3 basic or untransfected cells (Fig 4A), indicating its promoter activity. Administration of 150 nM mithramycin 1 hr before the transfection significantly reduced the promoter activity, expressed as 60–65% reduction in luciferase activity (Fig 4B). Mithramycin, induced a 10–15% reduction in luciferase activity in cells transfected with pTAL-Luc and pAP1-Luc, which do not contain GC-rich enhancer. The latter may be due to a reduction in overall activity of cells in the presence of mithramycin. Alternatively, this reduction may result from the inhibition of Sp1-dependent transcription of genes encoding proteins that activate AP1 and TATA. Nevertheless, the four-fold inhibition of luciferase activity in cells transfected with the *NDUFV2* promoter fragment, points to the involvement of Sp1 in the regulation its activity.

### Sp1 binds to the promoter region of NDUFV2

**Figure 5:**
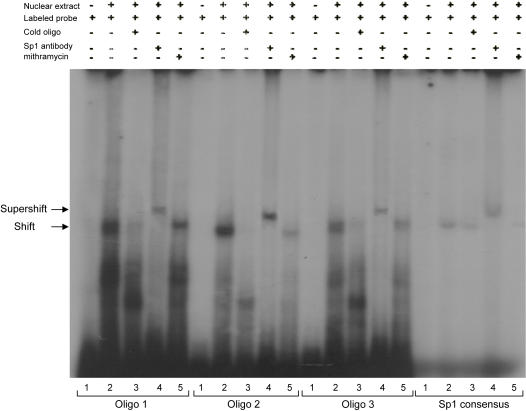
Sp1 binding to its three consensuses sequences in the 461 bp promoter sequence of *NDUFV2*. For electrophoretic mobility shift assay nuclear extracts of SH-SY5Y cells were incubated with the three double strand ^32^P-end labeled oligonucleotides (specified in Material and Method section), and a commercial ^32^P-end labeled oligonucleotides containing the consensus sequence for Sp1 (Sp1 consensus) in the presence (lanes marked 3) or absence (lanes marked 2) of cold competitors, or 150 nM mithramycine (lanes marked 5). For supershift assay, nuclear extracts were preincubated with Sp1 antibody prior to the addition of the appropriate oligonucleotide (lanes marked 4). Lanes marked 1 are free end-labeled probes.

GC-rich promoter elements are able to interact with a number of zinc finger transcription factors, including members of the Sp1 family and Kruppel-like factors [37], [38]. Moreover, mithramycin, which binds to GC-rich sites on DNA, is not a specific inhibitor of Sp1. Therefore, to test whether it is Sp1 that interacts with *NDUFV2*, we studied Sp1 binding to its 461 bp promoter region in the presence and absence of Sp1 specific antibody. Three putative Sp1 elements were synthesized as probes including the −120 to −147 region (I), the −171 to −200 region (II) and the −194 to −223 region (III). Gel mobility shift assay (Fig 5) revealed that all three probes formed DNA-protein complexes when incubated with SH-SY5Y nuclear extract (lane 2 for oligo 1–3) similar to that formed by double strand commercial probe containing the consensus sequence for Sp1 (lane 2 for Sp1 consensus). Addition of a 100 fold molar excess of the matching unlabeled double stranded probe (lanes marked 3), as well as 150 nM mithramycin (lanes marked 5) inhibited the formation of the DNA-protein complexes. Addition of Sp1 antibody to the binding reaction induced a supershifted band in all three probes, similar to their effect on the commercial Sp1 probe (lanes marked 4). These results further confirm the involvement of Sp1 in the regulation of *NDUFV2* promoter. The free, synthesized and commercial, [γ^32^P]-ATP end-labeled double stranded probes, are depicted in lanes marked 1.

## Discussion

Previously, we have reported schizophrenia and tissue specific alterations in the expression of complex I subunits, *NDUFV1* and *NDUFV2*, both at the level of mRNA and protein [13], [29]. This, together with numerous other studies reporting alterations in the expression of different groups of genes in schizophrenia [9], [39], may point to abnormal transcriptional regulation in the disorder. Indeed, in the present study we show that the expression of the ubiquitously expressed transcription factor Sp1 is altered in schizophrenia, in brain and in lymphocytes, as compared with normal controls. Specifically, Sp1 mRNA expression was down-regulated in the prefrontal cortex and the striatum, while up-regulated in the parieto-occipital cortex and in lymphocytes of schizophrenic patients. This opposite alteration in Sp1 expression in different brain regions is not unique for schizophrenia. Indeed, in Huntington's Disease (HD) it was previously reported that Sp1 levels were robustly increased in caudate postmortem tissues but decreased in the hippocampus [40].

The changes observed in Sp1 expression in schizophrenic subjects parallel the tissue specific pattern of alteration in *NDUFV1* and *NDUFV2* mRNA levels. In addition, a significant high correlation was observed between Sp1 and both *NDUFV1* and *NDUFV2* in normal subjects in all three brain regions. However, in schizophrenic patients the correlation between Sp1 and both *NDUFV2* and *NDUFV1* was distorted. In concordance with brain findings, a highly significant correlation between Sp1 and both subunits of complex I was observed in lymphocytes of normal subjects, which was eradicated in schizophrenic patients. Similarly, no correlation was observed between *NDUFV2* and *NDUFV1* in schizophrenic patients in all tissues examined. In normal subjects, however, a significantly high correlation was observed, as expected from the reported stoichiometry of 1 mol of each subunit for 1 mol of complex I [41]. These results suggest that Sp1 is involved in the regulation of the expression of both subunits of complex I.

It has been previously reported that Sp1 can be regulated at the level of transcription [14]. However, in order to find out whether the changes in mRNA levels are also expressed at the protein level, the active form of Sp1, we have analyzed Sp1 protein levels in brain specimens of schizophrenic patients and healthy subjects. Unfortunately, we ran out of protein samples from the prefrontal and the pariet-ocipital cortices, therefore we measured Sp1 in the striatum and the cerebellum. Protein levels of Sp1 in the striatum were significantly decreased by 42% in schizophrenic patients as compared to healthy subjects (2.39±0.31 vs. 1.38±0.27, p<0.0001, expressed as normalized OD values). Interestingly, in the cerebellum, where there was no significant difference in mRNA levels of both *NDUFV1* and *NDUFV2* between the schizophrenic and the normal groups, no significant change was observed in protein and mRNA levels of Sp1. Taken together, the results of this study in human subjects suggest that a defect in Sp1 transcriptional activity may play an important role in the abnormal expression of complex I subunits in schizophrenia.

One may argue that the alterations in the expression of complex I subunits and Sp1 may be due to the effect of antipsychotic medication. Indeed, it has been reported that chronic treatment with antipsychotic drugs affects the expression of many genes including immediate early genes, genes related to DA metabolism and genes involved in lipid biosynthesis [42]–[45]. However, in the present study medication, taken as a covariate, did not affect the significance of differences observed between the patients and the healthy subjects, in mRNA levels of both subunits of complex I and of Sp1, in all brain regions and in lymphocytes. Moreover, in our previous studies antipsychotic medication had no effect on the alterations in mRNA and protein levels of complex I subunits in postmortem brain specimens of antipsychotic treated schizophrenic as well as bipolar patients [13], [29]. Another confounding factor in postmortem brain studies, which has been extensively addressed, is the pH of the samples [46]–[48]. Although the mechanism by which pH affects the expression of genes is still unclear, it has been reported that the pattern of gene expression differs between low and high pH-brain specimens [46], [47]. However, in our study adding pH as a covariate did not change the disease related effects on the complex I subunits and on Sp1, similar to its lack of effect on the disease related differences in other genes such as glucocorticoid receptor, phosphorylated and non-phosphorylated cytosolic protein kinase Cε and kainate receptor 2 [49]. We therefore assume that disrupted Sp1 transcriptional activity is associated with schizophrenia pathology rather than its treatment.

Further evidence supporting the involvement of Sp1 in the transcription of the schizophrenia relevant genes, is the ability of mithramycin, which inhibits Sp1 binding to DNA GC-rich sites, to inhibit the expression of those genes. Mithramycin induced a time dependent decrease in mRNA and protein levels of complex I subunits *NDUFV1, NDUFV2* and *NDUFS1*, as well as of reelin, which was arbitrarily chosen as a gene known to be modulated by Sp1 and repeatedly reported to be abnormally expressed in schizophrenia [19], [50]. In our previous studies, the expression of *NDUFS1* was not significantly altered in schizophrenia in the prefrontal and the parieto-occipital cortices and in lymphocytes. However, in the cerebellum there was a significant reduction in *NDUFS1* (unpublished data). Moreover, it was reported that in a subgroup of early onset schizophrenic patients, *NDUFS1* was abnormally expressed in their lymphocytes [51]. These findings can be related to Sp1 being differentially subjected to endogenous or exogenous environmental stimuli [14]–[17], and possibly to the involvement of additional transcription factors in the expression of *NDUFS1 in vivo*.

In order to further study whether Sp1 plays a role in the regulation of genes abnormally expressed in schizophrenia, we chose the *NDUFV2* subunit of complex I, which was the most substantially affected in schizophrenia in both brain and periphery. First, we identified a promoter-like activity in the 461 bp genomic fragment of the 5′-flanking sequence upstream to the translation initiation ATG. The involvement of Sp1 in the activation of this promoter sequence, which contains three GC-rich elements, was then demonstrated by the robust reduction in its promoter activity in the presence of mithramycin.

GC-rich promoter sequences are able to interact with a number of zinc-finger transcription factors, including other members of Sp, Kruppl like factors, and TGF-β-inducible early gene families [18], [37], [38], [52]. Therefore, reduced expression as well as reduced promoter activity following mitramycin treatment may be due to other zinc-finger transcription factors. Therefore, competition gel shift assays were performed. GC-boxes are the only putative binding site in the *NDUFV2* TATA-less promoter sequence. Competition gel shift assays in the presence and absence of mithramycin, as well as supershift assays, have confirmed that Sp1 is one of the nuclear factors involved in the regulation of *NDUFV2* promoter. However, it is possible that other members of the zinc-finger transcription factors and of the Sp family will compete for binding of the three GC-rich sequence of the *NDUFV2* promoter, specifically Sp3 and Sp4, which share great homology with Sp1 and are able to recognize GC-box with identical affinities [14]. Further studies are needed to determine whether these zinc-finger transcription factors, in general, and the other Sp family members, in particular, are of relevance to the abnormalities observed in the other subunits of complex I implicated in schizophrenia.

Sp1 is a ubiquitously expressed transcription factor and is essential for the basal transcription of many genes containing the Sp1 binding site(s). However, evidence has accumulated supporting the specificity of Sp1 regulation. Alterations in its abundance were observed under various conditions such as during development, increased calcium, viral infection and hypoxia [14], [16], [53]. In addition, many studies have shown that Sp1 can play a role in the regulation of certain genes in response to specific signals. Thus, Sp1 activity can be regulated by diverse factors including high glucose and glucosamine, growth factors such as TGF-β and intracellular signaling such as cAMP, PKC and ERK, as well as by interaction with other nuclear factors [14]–[17], [52], [54], [55]. A study investigating the effect of such factors on Sp1 transcriptional activity and on the expression of complex I subunits as well as on other genes implicated in schizophrenia, is underway. In conclusion, the results of the present study, demonstrating disrupted expression of Sp1 associated with parallel impairments in complex I subunits, for which Sp1 is probably a transcription factor, suggest a key role for Sp1 in the pathogenesis of schizophrenia. The involvement of Sp1 in the regulation of many genes currently implicated in schizophrenia, together with the duality of Sp1 having a ubiquitous as well as environmental and intracellular specific signal regulated transcriptional activities, concur with the multi gene abnormality and symptom heterogeneity observed in schizophrenia.
